# Lung ultrasound score predicts outcomes in patients with acute respiratory failure secondary to COVID-19 treated with non-invasive respiratory support: a prospective cohort study

**DOI:** 10.1186/s13089-024-00365-6

**Published:** 2024-03-08

**Authors:** Mauro Castro-Sayat, Nicolás Colaianni-Alfonso, Luigi Vetrugno, Gustavo Olaizola, Cristian Benay, Federico Herrera, Yasmine Saá, Guillermo Montiel, Santiago Haedo, Ignacio Previgliano, Ada Toledo, Catalina Siroti

**Affiliations:** 1 Respiratory Intermediate Care Unit, Juan A. Fernandez Hospital, Av. Cerviño 3356, Buenos Aires, C1425 CABA Argentina; 2 Department of Medical, Oral and Biotechnological Sciences, University of G. d’ Annunzio, Chieti-Pescara, Italy; 3 Healthcare Unit Dr. Cesar Milstein, Buenos Aires, Argentina; 4 Police Medical Complex Churruca-Visca, Buenos Aires, Argentina; 5 Dr. Antonio A Cetrángolo Hospital, Buenos Aires, Argentina; 6 Bernardino Rivadavia Hospital, Buenos Aires, Argentina; 7 Rehabilitation and Respiratory Care Section, Italian Hospital of Buenos Aires, Buenos Aires, Argentina

**Keywords:** COVID-19, Acute respiratory failure, Ultrasonography, Non-invasive ventilation

## Abstract

**Background:**

Lung ultrasound has demonstrated its usefulness in several respiratory diseases management. One derived score, the Lung Ultrasound (LUS) score, is considered a good outcome predictor in patients with Acute Respiratory Failure (ARF). Nevertheless, it has not been tested in patients undergoing non-invasive respiratory support (NIRS). Taking this into account, the aim of this study is to evaluate LUS score as a predictor of 90-day mortality, ETI (Endotracheal intubation) and HFNC (High Flow Nasal Cannula) failure in patients with ARF due to COVID-19 admitted to a Respiratory Intermediate Care Unit (RICU) for NIRS management.

**Results:**

One hundred one patients were admitted to the RICU during the study period. Among these 76% were males and the median age was 55 (45–64) years. Initial ARF management started with HFNC, the next step was the use of Continuous Positive Airway Pressure (CPAP) devices and the last intervention was ETI and Intensive Care Unit (ICU) admission. Of the total study population, CPAP was required in 40%, ETI in 26%, while 15% died. By means of a ROC analysis, a LUS ≥ 25 points was identified as the cut-off point for mortality(AUC 0.81, OR 1.40, 95% CI 1.14 to 1.71; *p* < 0.001), ETI (AUC 0.83, OR 1.43, 95% CI 1.20 to 1.70; *p* < 0.001) and HFNC failure (AUC 0.75, OR 1.25, 95% CI 1.12 to 1.41; *p* < 0.001). Kaplan-Meier survival curves also identified LUS ≥ 25 as a predictor of 90-days mortality (HR 4.16, 95% CI 1.27–13.6) and 30 days ETI as well.

**Conclusion:**

In our study, a ≥ 25 point cut-off of the Lung Ultrasound Score was identified as a good outcome prediction factor for 90-days mortality, ETI and HFNC failure in a COVID-19 ARF patients cohort treated in a RICU. Considering that LUS score is easy to calculate, a multicenter study to confirm our findings should be performed.

**Supplementary Information:**

The online version contains supplementary material available at 10.1186/s13089-024-00365-6.

## Introduction

The hallmarck of the novel SARS-CoV-2 (Severe Acute Respiratory Syndrome Coronavirus 2) infection Coronavirus Disease 2019 (COVID-19), is acute respiratory failure (ARF) due to interstitial lung inflammation. Its incidence is about 19% and 5% of the infected patients will need intensive care support [1]. This kind of management could be performed at an Intensive Care Unit (ICU) or in a stepup unit as a Respiratory Intermediate Care Unit (RICU). RICUs have the capabilities to perform non-invasive respiratory support (NIRS) until patients improve or deteriorate and need ICU admission. Their development during COVID 19 pandemics allowed ICU beds saving and were very cost effective [2–4], despite controversies about the initial ARF management [3].

Several studies highlighted the role of lung imaging in COVID 19 ARF diagnosis [5–7] comparing chest computed tomography (CT), conventional chest x-rays and real-time reverse transcription-polymerase chain reaction (RT-PCR). Their sensitivity was 98%, 69% and 71% respectively, allowing an accurate diagnosis in RT-PCR negative patients with CT findings compatible with COVID-19 ARF. However, CT performance has its limitations, taking into account the cumbersome process of patient transferring, including virus spreading, and the ionization risks, which limits its liberal use [8, 9]. The concept of point-of-care ultrasound (POCUS) is the use of bedside ultrasound by non-radiologist physicians in order to make diagnoses, guide treatments or to perform invasive procedures safely. Lung POCUS proved to be very accurate in lung diseases diagnosis, inexpensive, repeatable, widely available and non-ionizing [10, 11]. Lung Ultrasound (LUS) score is a semi quantitative score that measures lung aeration loss in several pathologic conditions (12). LUS score provides risk stratification including mortality and indication of invasive mechanical ventilation (IMV) [11–13]. One of the most important critical points in the ARF management is the risk of delayed endotracheal intubation (ETI), which could be responsible for a worse outcome [14]. LUS score appears as an interesting option to identify patients in whom NIRS fails or has failed. A pilot study [15] seems to point in that direction. The aim of this study is to evaluate LUS usefulness as an outcome predictor for COVID-19 ARF patients treated with NIRS in a RICU.

## Materials and methods

Study design and setting: prospective cohort study of COVID-19 ARF patients admitted to the Fernandez Hospital RICU from June 2020 to February 2021. Institutional review board reviewed the protocol and authorized prospective data collection (Code register: ID #2263).

Primary study endpoint was to identify LUS cutoff point for 90-days mortality. Secondary endpoints were to identify LUS cut-off point for HFNC failure identification and ETI indication. HFNC failure was defined as the need to switch to CPAP devices to maintain oxygenation. NIRS was performed under a strict protocol [16] that is available online as supplementary content, as well as statistical data analysis.

### Patients

Consecutive COVID-19 ARF patients admitted to RICU were included. Patients with advance directives (do not intubate or do not resuscitate) and pregnant women were excluded. A 12 h HFNC trial at 60 L/min and F_i_O_2_ to maintain SpO_2_ between 92 and 96% was initiated if one of the following criteria was met: P_a_O_2_/F_i_O_2_ (P/F) ≤ 200, supplemental oxygen requirement ≥ 10 L/min, respiratory rate (RR) ≥ 30/min with or without accessory muscles usage (eFigure 1). Awake prone position was used as an adjuvant therapy. Patients were considered responsive if RR was < 30/min and SpO_2_ increased > 94% with F_i_O_2_ < 0.6% after the 12 h trial. In non-responders patients, NIRS was switched to CPAP.

ETI indication was performed if two of the following signs of ARF worsening were present: lack of improvement or worsening oxygenation, respiratory rate above 40/min, lack of improvement of signs of respiratory muscle fatigue, development of copious tracheal secretions, acidosis with a pH < 7.35, or intolerance to CPAP. The need for ETI was also established by the presence of one of the following criteria: hemodynamic instability (systolic blood pressure < 90 mmHg, mean blood pressure < 65 mmHg or requirement for vasopressor support), deterioration of neurologic status with a Glasgow Coma Scale below 12 points.

### Data collection

After selection, informed consent was granted. The following variables were collected: age, sex, body mass index (BMI), comorbidities, SOFA(Sequential Organ Failure Assessment), APACHE II (Acute Physiology and Chronic Health Evaluation), NEWSII (National Early Warning), day of illness, P/F, ROX index (Respiratory rate-OXygenation) defined as the ratio of oxygen saturation (SpO_2_)/fraction of inspired oxygen (F_i_O_2_) to RR at different times (2, 6, 12, 24 and 48-h) and LUS at admission.

### LUS protocol

**Fig. 1:**
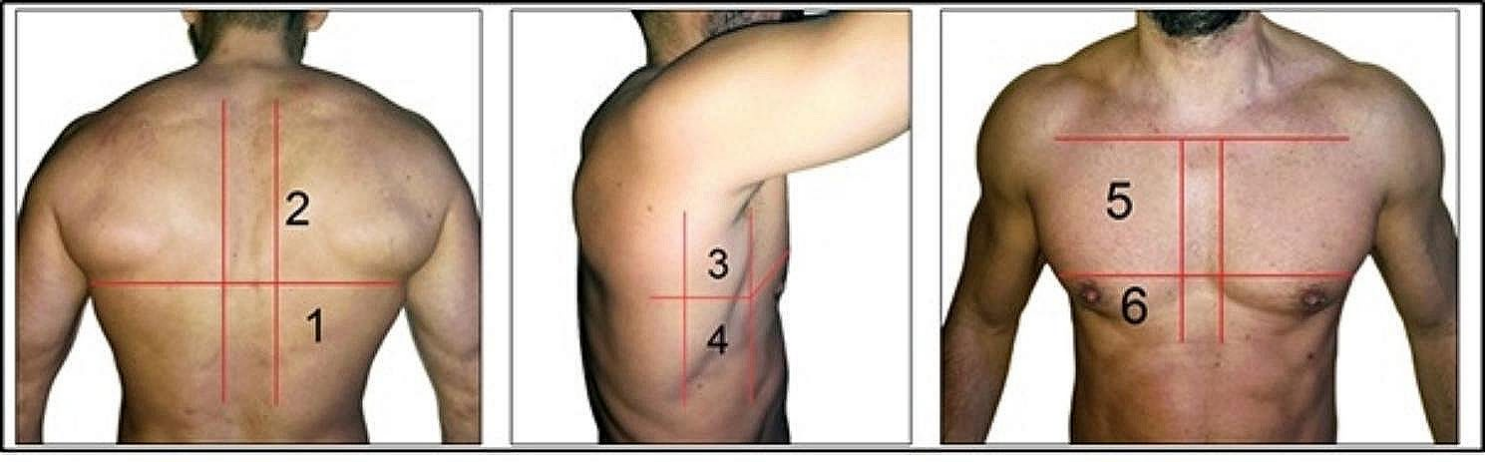
Region of interest for lung ultrasound score

Four Respiratory Therapists certified in lung POCUS by the Argentinean Association of Kinesiology and trained in LUS for 2 months prior the study, performed all the ultrasound measurements within 24 h of RICU admission. The exploration was performed by dividing the thorax into 12 zones, delimited by the parasternal line, the anterior axillary line, the posterior axillary line and a paravertebral zone on each side. The upper and lower reference is given by the perpendicular line to the previous ones, at the level of the nipples. Exploration technique is developed in Fig. 1. A 3.5-5 Hz convex probe was used to explore the thorax, placing the focus at the level of the pleural line (2–4 cm) and setting a depth of 8 to 10 cm. A scan was performed in each of the 12 zones in the longitudinal plane and the pattern of least aeration present in each zone was assessed. In case of requiring a better ultrasonic window and/or a better evaluation of the area, the transducer was placed in the transverse plane.

A semi quantitative score ranging, running from 0 to 3, was performed according to lung ultrasound findings: 0 = normal A lines, 1 = multiple separated B lines, 2 = coalescing B lines or light beam, 3 = consolidation. The aeration score is built by the sum of all the areas, with a minimum of 0 and a maximum of 36 according to the aeration loss. The following ultrasound devices were used; Philips Lumify® ultrasound machine (Philips Medical Systems, Bothell, WA, USA) with a convex transducer, a Sonoscape S6® ultrasound machine (Yizhe building, Yuquan Road, Shenzhen, 518,051, China) and a Chison ECO 1® (No.9, Xinhuihuan Road, Xinwu District, Wuxi, Jiangsu, China 214,028) were used for the measurements.

### Statistical analysis

**Fig. 2:**
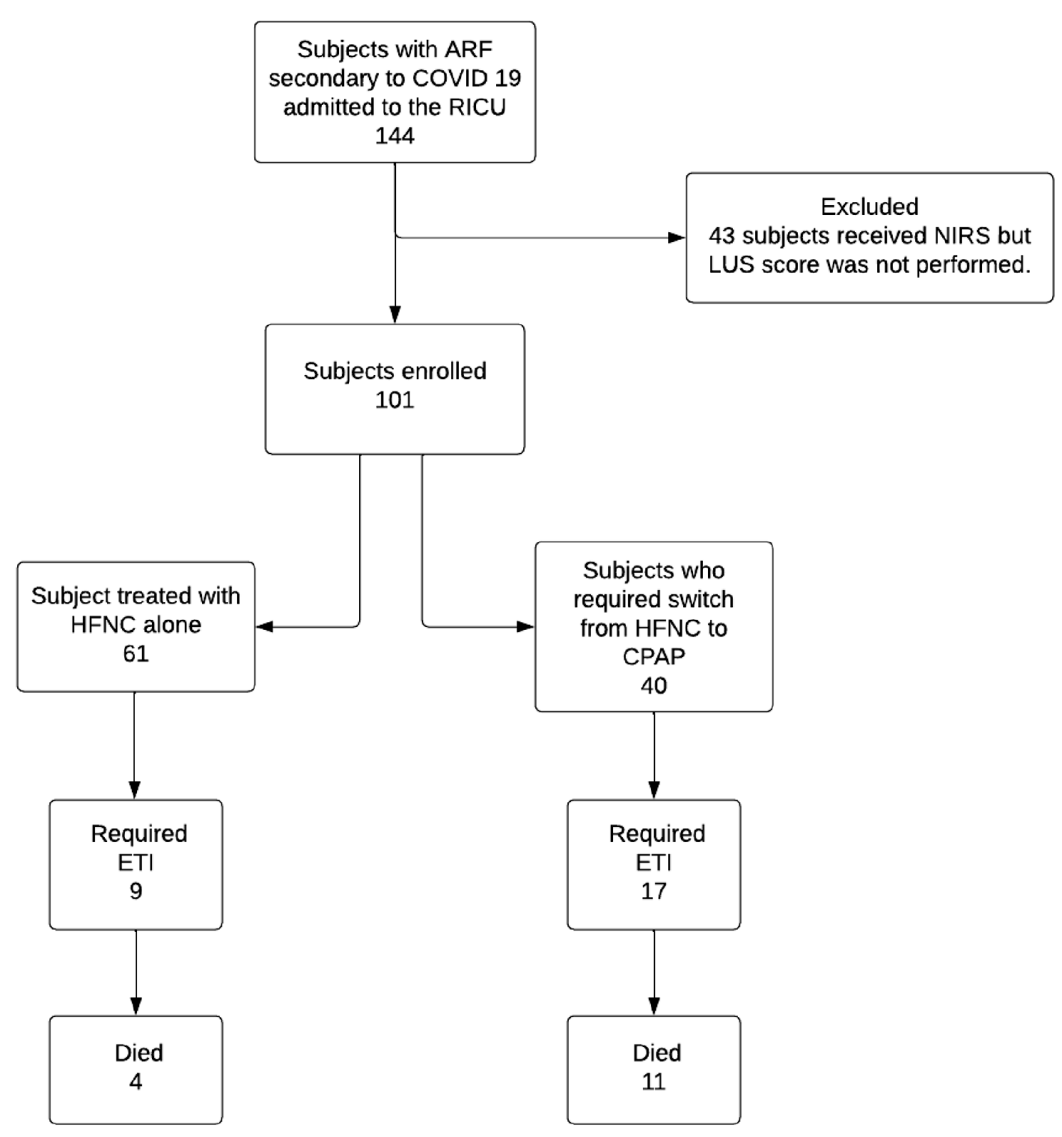
Patients allocation to non-invasive respiratory support. ARF: Acute Respiratory Failure; COVID-19: Coronavirus disease 19; HFNC: High-Flow Nasal Cannula; CPAP: Continuous Positive Airway Pressure; LUS: Lung Ultrasound

Sample size was not predetermined. Normality criteria was established by Schapiro Wilk test and according to it were presented as means ± standard deviations (± SD), medians and interquartile range (IQR). Categorical variables were presented as absolute values and percentages. Continuous variables were compared using the student’s t-test or the U-Mann Whitney test, as appropriate. For categorical variables, chi-square tests were used (Figure 2).

A Receiver Operating Characteristic (ROC) curve analysis was performed accorded to LUS findings on respect of primary and secondary outcomes. The area under the curve (AUC) was calculated to quantify the differences. LUS sensitivity and specificity were determined and the cut-off point corresponded to the maximum of the Youden’s index.

Kaplan-Meier curves were used for survival analysis and ETI incidence. In order to identify factors associated with the likelihood of in-hospital mortality, we fit a multivariable logistic regression model with mortality as the dependent variable. A priori selected variables were those considered of clinical relevance as well as variables that were significantly associated with the outcome in the bivariate analysis (at a p-value threshold of 0.2 or less). We report odds ratios (OR) with their associated 95% confidence intervals (CI). A p-value < 0.05 was considered statistically significant. Statistical analyses were performed using the Statistical Package for the Social Sciences (SPSS) version 26.0 (IBM Corporation, Armonk, NY, USA).

## Results

During the study period 144 consecutive patients were admitted. Among them 101 patients met the inclusion criteria, 76% were men with a median of 55 years old (45–64). Clinical and laboratory baseline characteristics are summarized in Table 1.

**Table 1:** Clinical and demographic characteristics

Characteristics	Survivor (*n* = 86)	Non-survivor (*n* = 15)	p-value
Age in years, median (IQR)	55 (45–63)	61(51–67)	0.115
Male, n (%)	62 (61)	15 (39)	**0.019**
BMI, kg/m^2^, median (IQR)	28 (25–32)	28 (26–31)	0.586
Days of symptoms upon admission, median (IQR)	8 (6–10)	8 (7–10)	0.920
APACHE II, median (IQR)	8 (7–10)	11 (8–13)	0.035
SOFA, median (IQR)	4 (3–4)	5 (4–5)	**< 0.001**
NEWS II, median (IQR)	11 (9–13)	11 (10–13)	0.110
LUS at admission, points, median (IQR)	21 (19–25)	26 (25–27)	**< 0.001**
PaO_2_/FiO_2_ ratio (mmHg), at admission, median (IQR)	106 (95–137)	95 (79–105)	**0.012**
**Comorbidities**			
Diabetes, n (%)	5 (5)	0 (0)	1.000
Hypertension, n (%)	10 (9)	2 (1)	0.851
Asthma, n (%)	2 (2)	0 (0)	1.000
COPD, n (%)	1 (1)	0 (0)	1.000
Congestive heart failure, n (%)	3 (3)	2 (1)	0.158
**Non-Invasive Respiratory Support**			
High-Flow Nasal Cannula, n (%)	57 (66)	4 (26)	N/A
Switched to CPAP, n (%)	29 (33)	11 (73)	N/A
ROXi at 2-h, median (IQR)	8.04 (7.52–10.35)	8.03 (5.62–10.14)	0.538
ROXi at 6-h, median (IQR)	8.90 (7.08–10.66)	9.12 (6.60–10.70)	0.789
ROXi at 12-h, median (IQR)	9.30 (7.62–11.35)	7.75 (6.12-10.00)	**0.029**
ROXi at 24-h, median (IQR)	10.26 (8.17–14.29)	6.96 (5.78-9.00)	**< 0.001**
ROXi at 48-h, median (IQR)	10.26 (8.17–14.29)	4.43 (3.70–5.55)	**< 0.001**
**Laboratory test**			
D-Dimer, (µg/L), median (IQR)	348 (259–650)	390 (315–587)	0.497
C-Reactive Protein, (mg/L), median (IQR)	11 (6–15)	9 (6–19)	0.882
Ferritin, (µg/L), median (IQR)	819 (455–1363)	1182 (745–1500)	0.231
pH, median (IQR)	7.41 (7.40–7.44)	7,42 (7.41–7.43)	0.604
PaCO_2_ mmHg, median (IQR)	35 (32–37)	35 (34–38)	0.591
PaO_2_, mmHg, median (IQR)	85 (76–107)	72 (62–84)	**0.014**
HCO_3_ meq/l, median (IQR)	22 (21–24)	23 (21–24)	0.837

IQR: Interquartile range; BMI: Body mass index; APACHE II: Acute Physiology and Chronic Health Evaluation; SOFA: Sequential Organ Failure Assessment; NEWS II: National Early Warning Score; LUS: Lung Ultrasound Score; CPAP: Continuous positive airway pressure; ROXi: Respiratory Rate-Oxygenation index

N/A: not available

**Fig. 3:**
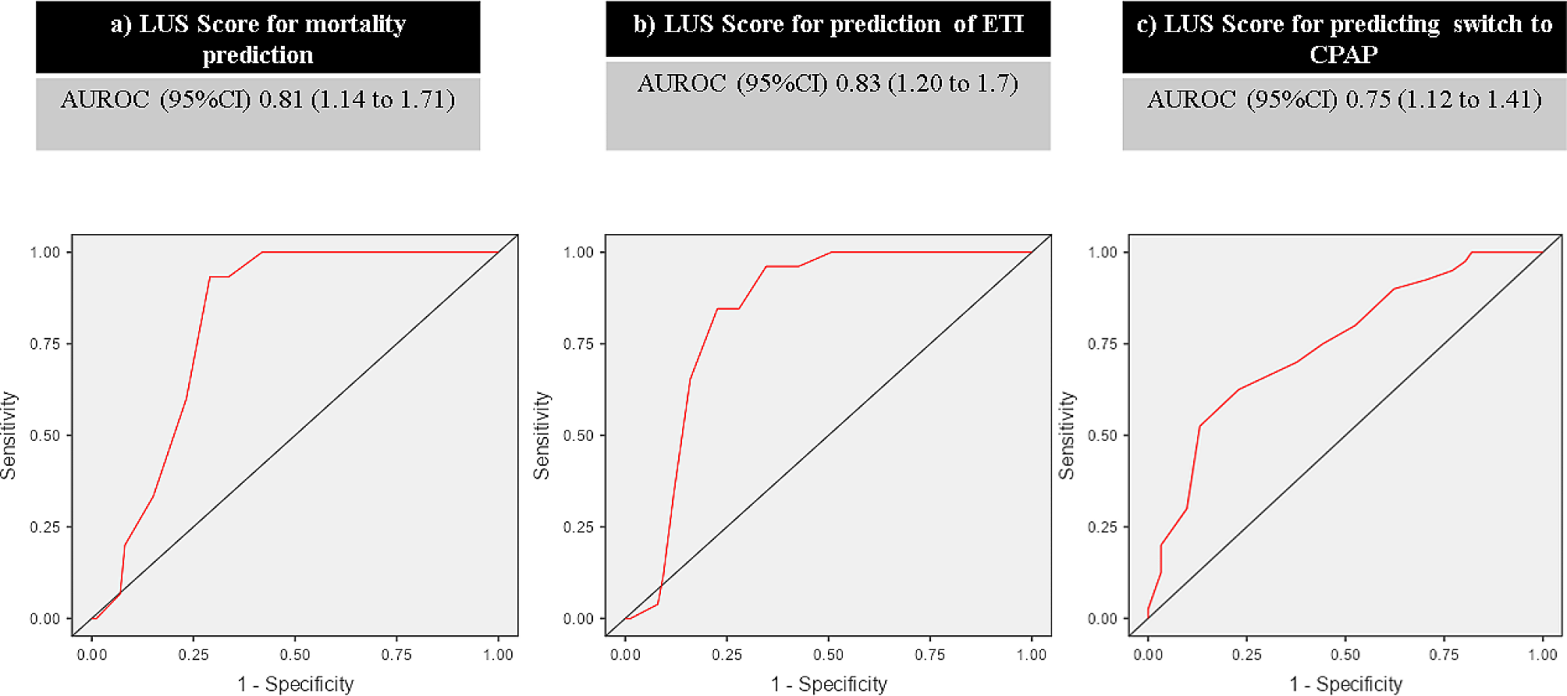
AUROC, LUS score in predicting (**a**) Mortality (**b**) ETI (**c**) Switch to CPAP. LUS score: Lung Ultrasound score; AUROC: Area under the receiver operating characteristic; ETI: Endotracheal intubation; CPAP: Continuous Positive Airway Pressure

HFNC 12-hours trial was performed in all subjects, 40% were switched to CPAP due to HFNC failure. Median LUS from HFNC responders were lower than the non-responders ones, LUS 21 (18–24) points vs. 26 (22–27) points, *p* < 0.001. ETI was indicated in 26% of the patients in a median of 2 days (2–3) after NIRS trial. A median LUS of 26 (25–27) was recorded in ETI patients and a median of 22 (20–27) in the CPAP responders (eFigure 2). Mortality rates of ETI patients was 57%, these 15 patients had greater LUS than the survivors (LUS 26 [25–27] points vs. 21 [19–25] points, *p* < 0.001). To find out LUS cut-off at different outcomes ROC curves and AUC were performed (Table 2). A 25 points cut-off was also predictive for mortality (AUC 0.81, OR 1.40, 95% CI 1.14 to 1.71; *p* < 0.001), ETI (AUC 0.83, OR 1.43, 95% CI 1.20 to 1.70; *p* < 0.001) and for switch from HFNC to CPAP (AUC 0.75, OR 1.25, 95% CI 1.12 to 1.41; *p* < 0.001), as shown in Fig. 3.

**Table 2:** Lung ultrasound score at admission and outcome cut-off

	LUS score cut-off	Sensitivity (%)	Specificity (%)	PPV (%)	NPV (%)
Discharge alive	≤ 23	100	58	29	100
Dead	**≥** 25	93	70	35	98
Non intubated	≤ 21	100	49	41	100
Intubated	**≥** 25	84	77	56	93
Switch to CPAP	**≥** 25	62	77	60	75

CPAP: Continuous positive airway pressure

**Fig. 4:**
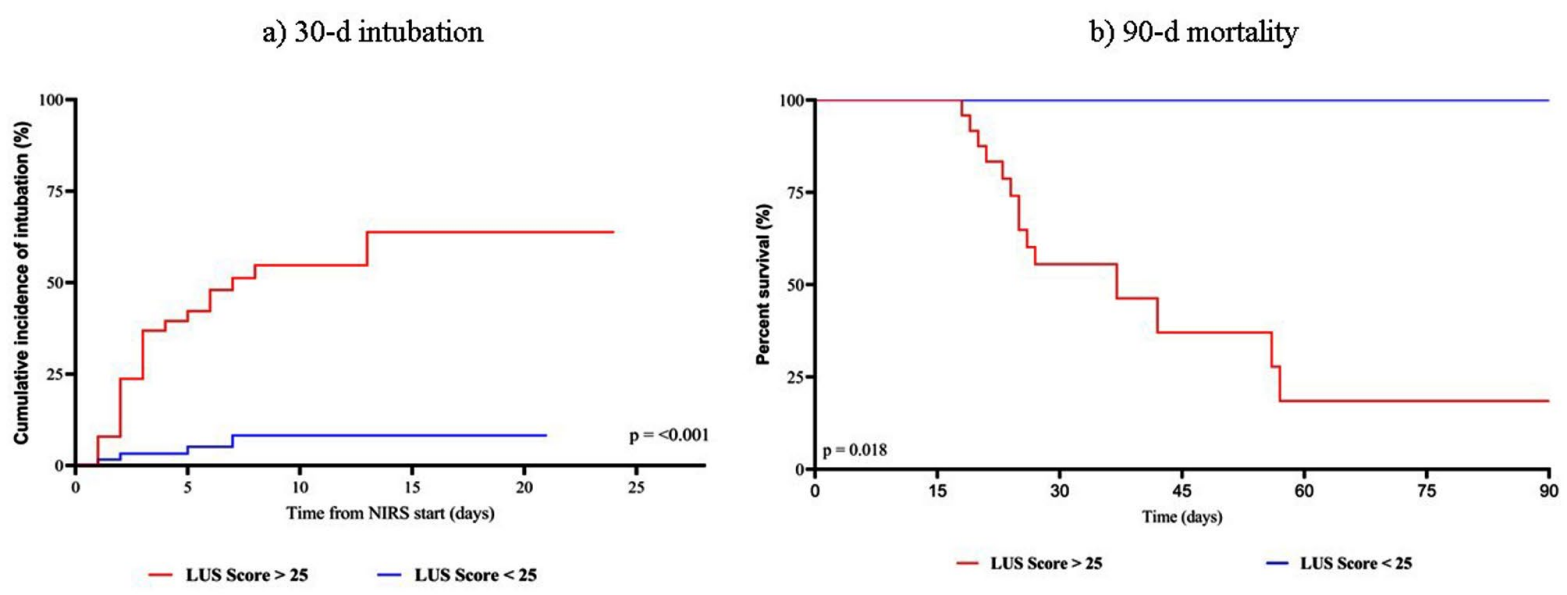
Kaplan-Meier plots of time-to-event data for the ETI (left) and mortality (right). NIRS: Non-Invasive Respiratory Support; LUS score: Lung Ultrasound Score, ETI: Endotracheal intubation

In Fig. 4 Kaplan Meier plot shows that a LUS ≥ 25 points patients have an increased 90-days risk of death (HR 4.16, 95% CI 1.27–13.6) and a higher ETI rate at 30 days (HR 9.28, 95% CI 4.25–21.4). In a multivariate logistic regression, SOFA score and LUS at admission were associated with risk of death (eTable 1). A significant inverse negative correlation was found between the LUS score and the ROX index at 12, 24 and 48 h (eFigure 3).

## Discussion

In this cohort of 101 COVID-19 ARF patients admitted to a RICU and treated with a strict NIRS protocol, LUS was a good predictor of 90-day mortality, switch from HFNC to CPAP and ETI requirement.

De Alencar et al. [17] found that LUS ≥ 26 predicts mortality during admission with an AUC 0.72 and 90% specificity. This was also published by Brahier et al. [18] with an AUC 0.76. Bonadia et al. [11] found that LUS measured on admission did not predict ICU requirement but had a good correlation with mortality. Lichter [19] and Sosa [20] arrived at the same conclusion. These findings point out that LUS score may be an important risk stratification tool for patients with COVID-19 ARF [21].

Regarding HFNC failure and the switch to CPAP, LUS score could be useful in its early identification by means of the lower lung aeration, that would reflect the higher PEEP levels needs than those administered with HFNC. Baciarello et al. showed that the P/F ratio was inversely related to the LUS score, decreasing by approximately 3.66 mmHg for each additional point in LUS score [22]. Patients with lower ROX index show worse outcomes, especially after 12 h [23]. In our Spearman correlation test, the ROX index at 24 and 48 h was inversely related to the LUS score, the higher the LUS score, the lower ROX index. This could support the idea that higher PEEP levels (NIV/CPAP or IMV) should be taken into account when scores are ≥ 25.

Biasucci et al. found that a high LUS score (> 12 points) at admission to the emergency department was associated with non-invasive ventilation (NIV) failure and the need of IMV (AUC 0.94, 95% CI 0.83–0.99; sensitivity 88%, specificity 93%). Analogous to our results, those who used HFNC successfully had significantly lower LUS scores compared to those who required mechanical ventilation [invasive or not] (9, IQR 8–10) vs. (12 IQR 8–14), respectively (both *P* < 0.01) [15]. We hypothesize that the differences in LUS score values (HFNC versus mechanical ventilation requirement [invasive or not]) between our study and Biasucci et al. are due to the fact that their protocol evaluated 6 zones (three on each side). However, this probably reinforces the idea that an abbreviated ultrasonographic assessment protocol can be performed without compromising predictive power.

In our study, patients requiring ETI and those needing switch from HFNC to CPAP had the same LUS score cut-off (≥ 25 points). In fact of 40 patients who required CPAP, 17 (43%) required endotracheal intubation (LUS 26 [26–27] points) and 11 (65%) of them died. On the other hand, 23 of 40 patients avoided intubation even though 8 of them (35%) had a LUS > 25. This suggests that with a LUS ≥ 25 the need for MV (invasive or non-invasive) should not be avoided. If non-invasive ventilation is used, strict and comprehensive monitoring is necessary in order to not delay ETI and detect the CPAP responder. With respect to the 8 patients (switched to CPAP) who did not require ETI despite LUS > 25, one of the hypotheses about this is that ETI is not defined by pulmonary compromise severity alone. Rather, variables related to respiratory center response to this compromise may define ETI (increased work of breathing, impaired gas exchange and RR). Likewise, other variables such as hypoxemia mechanisms with predominant vascular involvement, intolerance to treatment, hemodynamic instability or medical criteria are also not reached by the LUS score. According to our protocol, when CPAP was established, the patient was classified as “ventilatory alert” and if there was no real improvement, ETI was performed.

Another possible explanation for patients with LUS score > 25 but different outcomes (CPAP vs. ETI), is that the LUS score identifies the presence of consolidations with a score of 3 points. However, it does not take into account the extent of consolidations, so that two different images categorized as consolidations (3 points) could have different clinical repercussions. Our experience in terms of ETI rate is similar to that reported by Franco et al. (~ 30%) and Grieco et al. (~ 40%) [4, 24].

Our study has several limitations. First of all, this was a single center experience, which does not allow for any generalization of the results. The sample size was not calculated, so it is not defined whether it is exact or small. Finally, an inter or intra observer test was not carried out. The prospective evaluation, the consecutive enrollment and the pre-established NIRS protocol are some of our study strengths. Likewise, we believe that the value of this study lies in the fact that it confirms previously reported results about the role of LUS as a pulmonary severity stratifier, as a tool with great potential prognostic value, while addressing less studied outcomes, such as the need for ETI during NIRS treatment, in a larger cohort of patients. Although the robust statistical findings support the usefulness of LUS, further large multicenter studies are needed.

## Conclusions

LUS score is a simple tool that can be assessed bedside in COVID-19 ARF patients treated in a RICU setting, by means of a NIRS protocol. In our population a LUS ≥ 25 points predicts 90-days mortality risk and ETI requirements at 30 days, as well as HFNC failure. We believe that our findings could be the cornerstone for a large multicenter prospective observational trial.

### Electronic supplementary material

Below is the link to the electronic supplementary material.

Supplementary Material 1

## Data Availability

The data sets used and/or analyzed during the current study are available from the corresponding author on reasonable request.
