# Supplementary material for: Lung ultrasound score predicts outcomes in patients with acute respiratory failure secondary to COVID-19 treated with non-invasive respiratory support: a prospective cohort study
Source: Ultrasound J. 2024 Mar 8;16:20. doi: 10.1186/s13089-024-00365-6 (PMC10923765; doi:10.1186/s13089-024-00365-6)
Supplement: Supplementary file 1 — Supplementary Material 1 [file 13089_2024_365_MOESM1_ESM.docx]

**Supplemental Online Content**

Lung Ultrasound Score predicts outcomes in patients with Acute Respiratory Failure secondary to COVID-19 treated with non-invasive respiratory support: A prospective cohort study. Mauro Castro-Sayat^1^, Nicolas Colaianni-Alfonso^1^, Luigi Vetrugno^2^, Gustavo Olaizola^3,7^, Cristian Benay^4,6^, Federico Herrera^1^, Yasmine Saá^1^, Guillermo Montiel^1^, Santiago Haedo^1^, Ignacio Previgliano^1^, Ada Toledo^1^ and Catalina Siroti^1^

**eTable 1.** Multivariate logistic regression for in-hospital mortality

**eFigure 1.** Decision-making protocol for the use of non-invasive respiratory support

**eFigure 2**. LUS score assessment at admission and; need for noninvasive respiratory support, need for orotracheal intubation and mortality.

**eFigure 3.** Spearman’s correlation for LUS at admission and ROXi at 12, 24 and 48-h

This supplemental material has been provided by the authors to give readers additional

information about their work.

**eTable 1.**

| **Variable** | **Odds ratio (95% CI)** | **p-value** |
| --- | --- | --- |
| SOFA | 4.32 (1.66-13.46) | 0.005 |
| APACHE II | 1.09 (0.83-1.46) | 0.131 |
| P_a_O_2_/F_i_O_2_ | 0.98 (0.95-1.00) | 0.543 |
| LUS score at admission | 1.61 (1.24-2.27) | 0.002 |
| SOFA: Sequential Organ Failure Assessment, APACHE II: Acute Physiology and Chronic Health Evaluation, LUS: Lung Ultrasound | | |

**eFigure 1.**

ARF: Acute Respiratory Failure; HFNC: High Flow Nasal Cannula; CPAP: Continuous Positive Airway Pressure; ICU: Intensive Care Unit.

**eFigure 2**.

Figure A) shows the LUS scores of the 61 patients who required HFNC alone (LUS 21 [18-24]) versus those 40 who required change to CPAP (26 [22-27]); (p<0.001). Figure B) illustrates the LUS scores of the 26 patients who required ETI (LUS 26 [25-27] versus the 75 (LUS 21 [18-24]) who avoided ETI; (p<0.001). Finally, Figure C) highlights the LUS scores of the 86 survivors (LUS (21 [19-25]) versus the 15 non-survivors (26 [25-27]); (p<0.001).

LUS score: Lung Ultrasound Score; HFNC: High Flow Nasal Cannula; CPAP: Continuous Positive Airway Pressure; ETI: Endotracheal intubation.

**eFigure 3.**

LUS score: Lung Ultrasound Score; ROX Index: Ratio OXygenation Index.


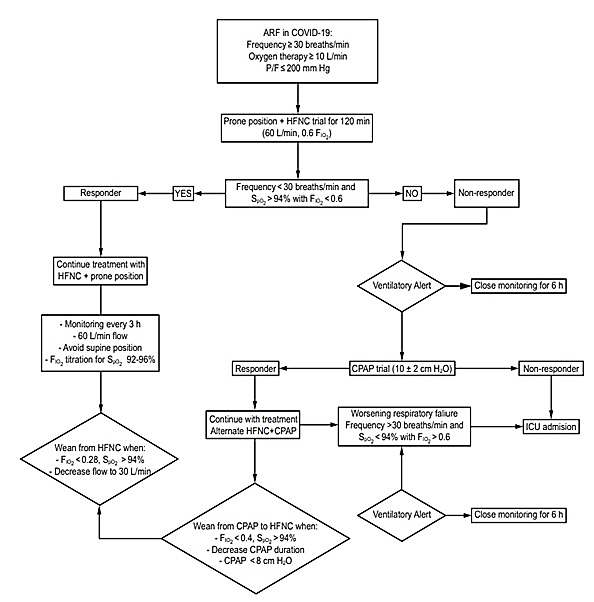


**eFigure 1.**


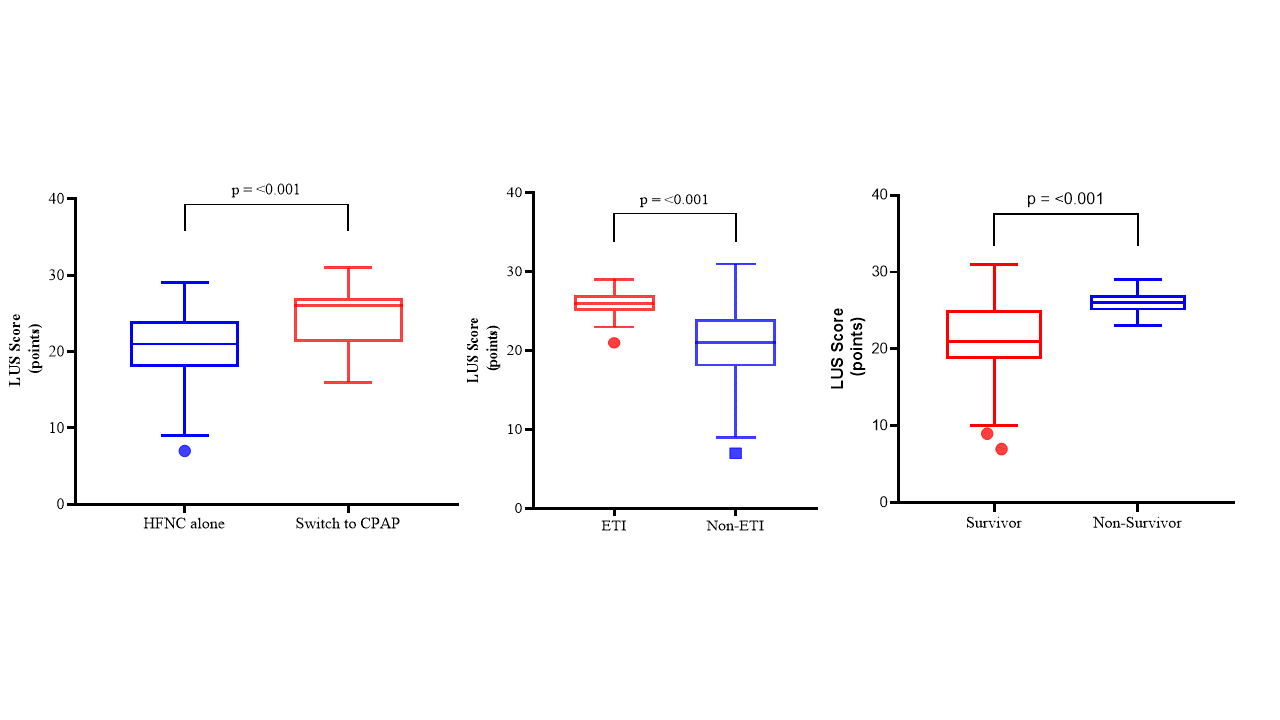


**eFigure 2.**

C)

B)

A)


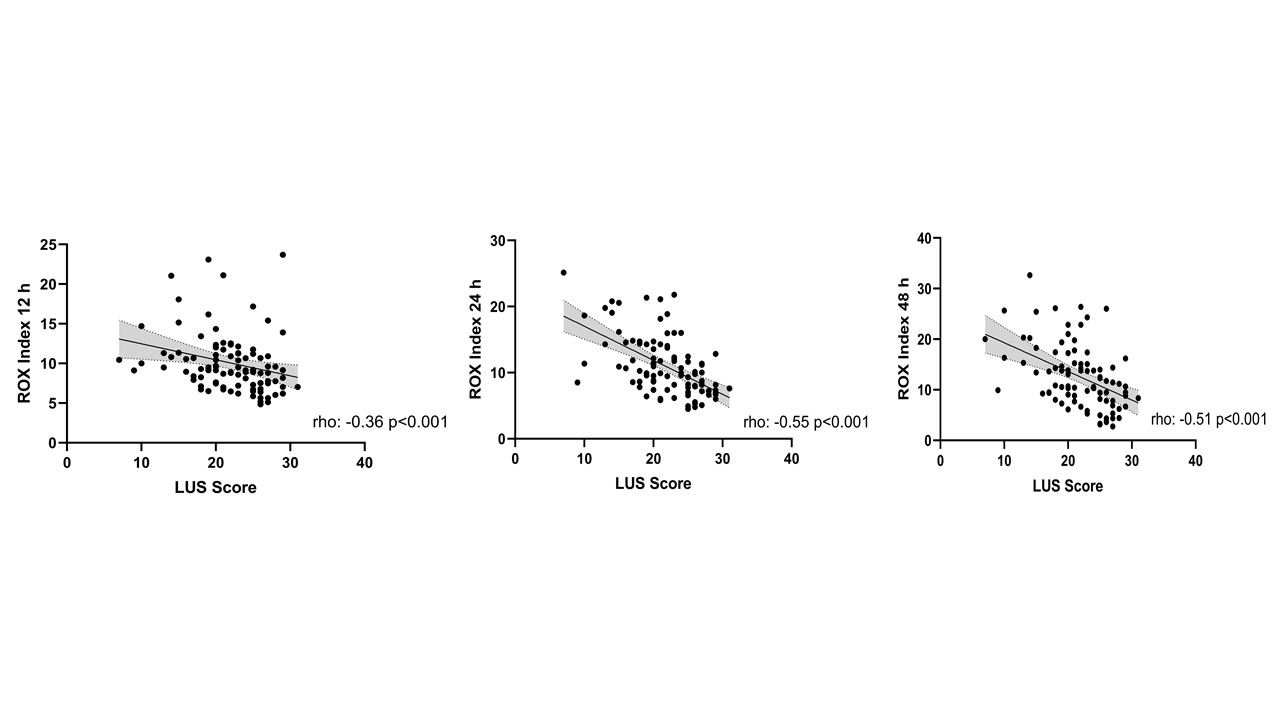


**eFigure 3.**
